# Magazine Notes

**Published:** 1898-11

**Authors:** 


### MAGAZINE NOTES.

*The Living Age*, in its issue for October 1st, is to begin a new serial story, translated for its pages from the French of Th. Bentzon (Mme. Blanc). The story is entitled "Constance" and it is the story of the life of a young girl. Important ethical questions, especially that of divorce, are touched upon, and the story has a high moral purpose. The translation is made by Mrs. E. W. Latimer, and is authorized by Mme. Blanc.

With the first number for October, *The Living Age*, the weekly eclectic magazine, which for more than fifty years has been a favorite with American readers, begins a new series and appears in a new and attractive dress, suggesting *The Atlantic Monthly* in the clear legibility of its page. The familiar cover is to be retained, but it has been newly engraved and otherwise modernized.

*The Living Age*, being a weekly magazine, suffers somewhat by comparison with the monthly magazines of the first class, if the comparison is made of single numbers. But *The Living Age* actually gives a larger amount of matter each month than any of the monthlies. Thus *Harper's Magazine* contains 172 pages each month; *The Century*, 160 pages; *Scribner's Magazine*, 128 pages; and *The Atlantic Monthly*, 144 pages; while *The Living Age* gives each month from 289 to 344 pages, according as there were four or five issues.

Florence Bell's "Plea for the Better Teaching of Manners," in *The Living Age* for October 1st, will be profitable to all who, as teachers or parents, have anything to do with the training of young people.

